# A Technical Review of Convolutional Neural Network-Based Mammographic Breast Cancer Diagnosis

**DOI:** 10.1155/2019/6509357

**Published:** 2019-03-25

**Authors:** Lian Zou, Shaode Yu, Tiebao Meng, Zhicheng Zhang, Xiaokun Liang, Yaoqin Xie

**Affiliations:** ^1^Shenzhen Institutes of Advanced Technology, Chinese Academy of Sciences, Shenzhen, China; ^2^Shenzhen College of Advanced Technology, University of Chinese Academy of Sciences, Shenzhen, China; ^3^Cancer Center of Sichuan Provincial People's Hospital, Chengdu, China; ^4^Department of Radiation Oncology, The University of Texas Southwestern Medical Center, Dallas, TX, USA; ^5^Department of Medical Imaging, Sun Yat-sen University Cancer Center, Guangzhou, China; ^6^Medical Physics Division in the Department of Radiation Oncology, Stanford University, Palo Alto, CA, USA

## Abstract

This study reviews the technique of convolutional neural network (CNN) applied in a specific field of mammographic breast cancer diagnosis (MBCD). It aims to provide several clues on how to use CNN for related tasks. MBCD is a long-standing problem, and massive computer-aided diagnosis models have been proposed. The models of CNN-based MBCD can be broadly categorized into three groups. One is to design shallow or to modify existing models to decrease the time cost as well as the number of instances for training; another is to make the best use of a pretrained CNN by transfer learning and fine-tuning; the third is to take advantage of CNN models for feature extraction, and the differentiation of malignant lesions from benign ones is fulfilled by using machine learning classifiers. This study enrolls peer-reviewed journal publications and presents technical details and pros and cons of each model. Furthermore, the findings, challenges and limitations are summarized and some clues on the future work are also given. Conclusively, CNN-based MBCD is at its early stage, and there is still a long way ahead in achieving the ultimate goal of using deep learning tools to facilitate clinical practice. This review benefits scientific researchers, industrial engineers, and those who are devoted to intelligent cancer diagnosis.

## 1. Introduction

Breast cancer threatens women's life worldwide. In the United States, it might cause an estimation of 0.25 million new cases of invasive breast cancer, 0.06 million new cases of noninvasive breast cancer, and 0.04 million deaths in 2016 [1]. This disease dramatically increases the health burden on those developing and underdeveloped countries [2]. Substantial clinical trial indicates that early detection and diagnosis of breast cancer can provide patients with more flexible treatment options and improved life quality and survivability [3]. Therefore, more and more attention is being paid to related fields, such as novel imaging modalities of ultrasound tomography [4] and breast tomography [5].

Mammography performs as a routine tool for breast cancer screening. It enables high-resolution perception of the internal anatomy of breast and helps the diagnosis of suspicious lesions [6]. Screening mammography scans the breast from the craniocaudal view and mediolateral oblique view, while diagnostic mammography acquires more images when symptoms, such as architecture changes and abnormal findings, are found on screening mammographic images. To date, screen film mammography (FM) has been the reference standard for use in breast cancer screening programs, while due to the demands of higher spatial resolution, digital mammography (DM) has been widely accepted. General rules exist for mammographic image interpretation. However, errors are unavoidable in clinic, and reasons are manifold. Above all, the difference of perceived visual appearance between malignant and benign lesions is unclear and consequently, how to quantify breast lesions with discriminative features is full of challenges. Moreover, it is still difficult to estimate the disease risk because of limited information and thus, healthy people might be turned into patients. Besides, work overload and fatigue further cause misinterpretation and overdiagnosis. Unfortunately, it is found that more than 70% of suggested biopsies are with benign outcomes during the diagnosis phase [7].

Computer-aided models for mammographic breast cancer diagnosis (MBCD) have been explored for over thirty years [8, 9]. It supports the decision making and helps the differentiation between malignant and benign lesions by providing additional information. Due to the facilitation of MBCD models, the diagnostic performance is enhanced regarding both sensitivity and specificity [10] and unnecessary examinations can be reduced in a cost-effective manner. It further benefits biopsy recommendations, follow-up treatments, and prognosis analysis. From a technical perspective, major MBCD models are consisted of feature extraction and lesion malignancy prediction. The former quantifies lesions with discriminative features and the latter builds the relationship between the features and its label, benign or malignant. Massive studies have devoted to the investigation of breast cancer diagnosis, ranging from using different modalities [11–13], to the analysis of subtle signs [14, 15] and to various technique exploration [16, 17]. Because of the easy accessibility of high-performance computing resources, millions of labeled data, and advanced artificial intelligence methods, convolutional neural network (CNN) has revolutionized image representation and benefited a broad range of applications [18], including but not limited to object recognition [19], visual understanding [20], and numerical regression [21, 22]. Quite different from conventional MBCD techniques, CNN attempts to integrate the feature extraction and lesion classification into a supervised learning procedure. The input of the CNN architectures is image patches of outlined lesion regions, and its output corresponds to the predicted lesion malignancy and intuitively, time and labor can be reduced in feature engineering. Meanwhile, CNN is pushing forward the technique upgrading in the field of medical imaging [23], medical physics [24, 25], medical image analysis [26–28] and radiotherapy [29, 30]. The research toward developing effective and efficient CNN-based MCBD models is still ongoing.

To the best of our knowledge, three review papers have been published regarding deep learning based breast cancer diagnosis. One concerns lesion detection and malignancy prediction using mammography, ultrasound, magnetic resonance imaging and digital tomosynthesis [31]. One focuses on mammography and histology image processing and analysis [32]. Meanwhile, it attempts to map the features/phenotypes between mammographic abnormalities and histological representation. The last one overviews deep learning in the detection and diagnosis of various kinds of cancers by using different imaging modalities [33]. In general, technical details in these review papers are not well delivered.

This paper also presents a review. It is dedicated to the technique of CNN applied in a specific application of MBCD, and it aims to provide clues on how to use CNN in intelligent diagnosis. The contributions of this review are summarized as follows. At first, this study is restricted to peer-reviewed journal publications and consequently, technical details and pros and cons of each model can be delivered. Furthermore, based on how to use CNN techniques, the MBCD models are broadly categorized into three groups. One is to design shallow models or to modify existing models for decreased time cost and medical instances for training; another is to make the best use of a pretrained CNN model by transfer learning and parameter fine-tuning; and the third is to take advantage of CNN models for feature extraction, while the differentiation between malignant and benign lesions is based on machine learning classifiers. At last, findings, challenges, and limitations are summarized, and some clues on the future work are also given.

The remainder of this paper is structured as follows.  Section 2 describes basic concepts regarding computer-aided diagnosis (CAD) and transfer learning.  Section 3 reviews CNN-based MBCD techniques, including the search strategy of the literature and technical details of involved models. And then, findings, challenges, and future focus are summarized in Section 4. In the end, Section 5 concludes this review.

## 2. Basic Concept of CAD Models

**Figure 1:**
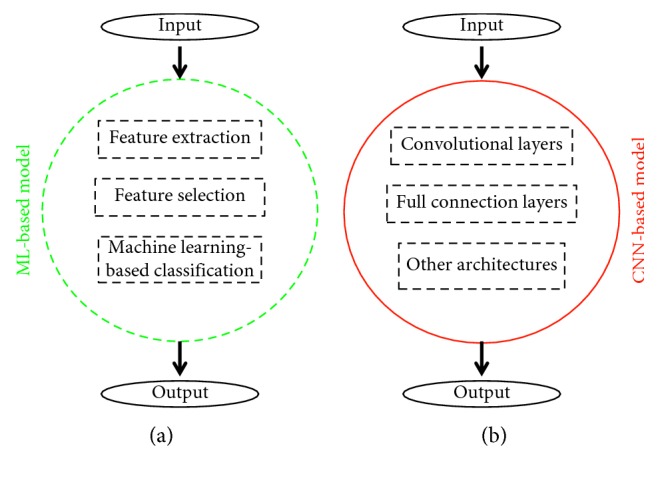
The diagram of the main flow chart of ML-based CAD (a) and major architectures of CNN-based CAD (b). The black dashed line indicates the blocks are modifiable. The green dashed line denotes each step in the ML-based model is interpretable, and the red solid line indicates the CNN-based model is data-driven when the architecture is fixed.

This section briefly describes the basic concepts of computer-aided diagnosis (CAD) and transfer learning. Specifically, Figure 1 shows the flow chart of machine learning- (ML-) based CAD and major architectures of CNN-based CAD. It should be noted that for diagnosis, a CAD model assumes the suspicious lesion regions have been accurately delineated and its purpose is to predict the malignancy of the input lesions.

### 2.1. Computer-Aided Diagnosis (CAD)

A CAD model can be used to provide additional information and support the decision making on disease diagnosis and cancer staging. It is different from a computer-aided detection model which aims to detect, localize, or segment suspicious regions. However, it should be noticed that a computer-aided detection model can be placed ahead of a diagnosis model for comprehensive analysis from the detection and localization to the diagnosis of suspicious regions.

#### 2.1.1. ML-Based CAD

A ML-based CAD model consists of feature extraction and machine learning-based classification as shown in the left of Figure 1, and feature selection is optional. Widely used features come from image descriptors that quantify the intensity, shape, and texture of a suspicious region [34]. Preferred machine learning classifiers are not limited to artificial neural network (ANN), support vector machine (SVM), k-nearest neighbors, naive Bayesian, and random forest (RF) [35]. Due to the emergency of radiomics [36–38], it should be noted that feature selection becomes more and more important and it aims to retrieve intrinsic features of suspicious lesions.

Mathematically, the procedure of using a pretrained ML-based CAD model to predict the malignancy of a lesion can be described as follows. First, an outlined suspicious region (*I*_*x*_) as the input is quantified with scalar variables (*E*(*I*_*x*_)) by using feature extraction (*E*). Then, feature selection (*S*) is employed to decrease the feature dimension and to retrieve informative features (*S*(*E*(*I*_*x*_))). In the end, the output of the label (*y*) of the lesion (*I*_*x*_) is predicted using machine learning classifiers can be formulated as *y*=*F*(*S*(*E*(*I*_*x*_))). For comprehensive understanding, overviews regarding machine learning and breast cancer diagnosis can be referred to [8, 9].

#### 2.1.2. CNN-Based CAD

**Figure 2:**
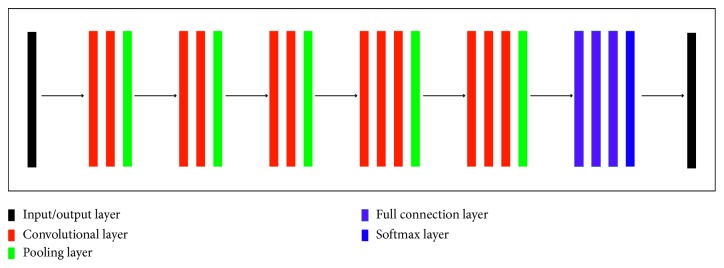
The architecture of VGG16. It consists of 13 convolutional layers, 3 full-connection layers, 5 pooling layers, and 1 softmax layer in addition to the input and output layers.

CNN models are computational models that are composed of multiple processing layers to retrieve features from raw data with multilevel representations and hierarchical abstraction [19]. As shown in the right of Figure 1, a general architecture of CNN models is made up of convolutional layers, full-connection layers, and pooling layers in addition to the input and output layers. Specifically, Figure 2 shows the architecture of VGG16 which consists of 13 convolutional layers, 3 full-connection layers, 5 pooling layers, and 1 softmax layer [39]. For further improvement in object classification, many techniques can be embedded, including nonlinear filtering, data augmentation, local response normalization, hyperparameter optimization, and multiscale representation [31, 32]. At present, widely used deep learning models include, but are not limited to, VGG [39], LeNet [40], AlexNet [41], GoogLeNet [42, 43], ResNet [44], YOLO [45], faster R-CNN [46] and LSTM [47].

Mathematically, the procedure of using a pretrained CNN-based CAD model for the prediction of lesion malignancy can be described as following. Given a suspicious region (*I*_*x*_), the output of a CNN-based model can be formalized as *y*=*F*(*I*_*x*_)=*f*_*n*_(*f*_*n*−1_(⋯(*f*(*I*_*x*_)))) where *n* stands for the number of hidden layers and *f*_*i*_ denotes the activation function in the corresponding layer *i*. Furthermore, how to design the architecture of deep learning models in addition to the comprehensive analysis and systematic methodologies of learning representation can be referred from [18, 19, 48].

It should be noted that CNN models are data-driven and can be trained end-to-end. The models enable the integration of feature extraction, feature selection, and malignancy prediction into an optimization procedure. Therefore, these retrieved features are not designed by human engineers but learned from the input data [19]. In general, remarkable performance of CNN-based CAD models comes from advanced computing hardware resource (i.e., graphic processing units and distributed computing system), open-source software, such as TensorFlow (https://www.tensorflow.org/), and open challenges based on millions of high-quality labeled images, such as ImageNet (http://www.image-net.org/). Its success also benefits from the novel design of architectures for deep learning, such as inception [43] and identity mapping [44].

### 2.2. Transfer Learning

**Figure 3:**
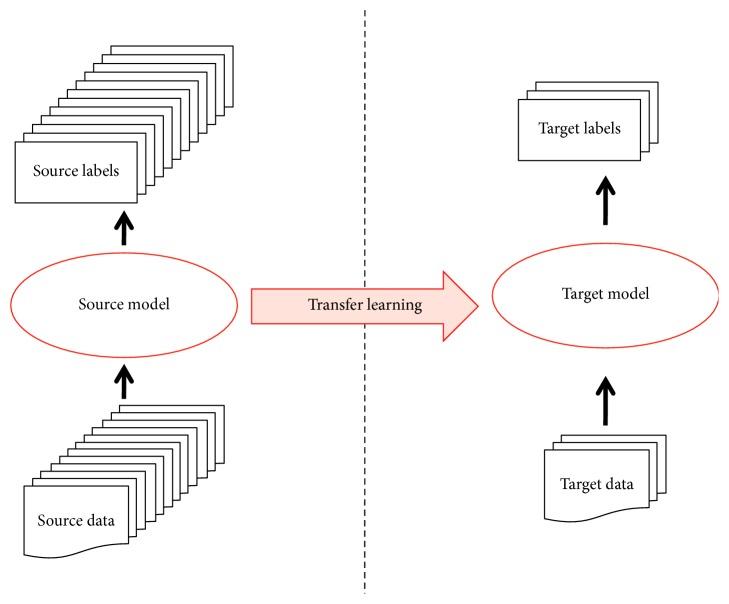
The diagram of knowledge transferred from the source domain to a different but related target domain. In the source domain, a model is trained with sufficient high-quality instances (data and labels) and transfer learning enables the model used in a related target domain. It relieves the requirement of huge amount of instances for the training of deep models in the target domain which is critically helpful in medical imaging field.

Transfer learning, or knowledge transfer, is more a machine learning strategy. It aims to reuse a model pretrained in the source domain as a starting point in a different but related target domain [49]. In the field of machine learning, an algorithm is typically designed to address one isolated task, while through transfer learning, the algorithm can be further adapted to a new task (Figure 3). It has several benefits using knowledge transfer. Above all, knowledge transfer enables the quality of the starting point in the target domain and thereby, promising results can be expected. Moreover, how to make use of a pretrained model is flexible. The model can be employed as a feature extractor for high-level representation of images and its parameters can be fine-tuned with target data. In addition, both time and cost can be reduced dramatically. Depending on computing resources, it takes about days to months training a deep model, while the time drops to hours when transferring this model for target applications. Thanks to the accessibility of pretrained deep models online, high-cost hardware seems unnecessary. Most importantly, transfer learning relieves the requirement of huge amount of instances for model training, which is critically helpful in medical imaging field. At present, the most popular object classification is based on the ImageNet [50] and without additional comments, pretrained CNN models are all denoted an initialization on the ImageNet in this study.

## 3. CNN-Based MBCD

This section firstly introduces the search strategy of literatures, involved databases and performance metrics. In the end, CNN-based MBCD methods are categorized into three groups based on the design and use of CNN models. This overview concentrates on peer-reviewed journal publications, and it provides technical details and pros and cons of CNN models.

### 3.1. Search Strategy for Literature Review

For the literature survey, IEEEXplore, Pubmed, ScienceDirect, and Google Scholar were used to search publications relating to CNN-based MBCD. The last update was at December 20, 2018. Keywords are “convolutional neural network,” “deep learning,” “mammography,” “breast cancer,” and “diagnosis”. Specifically, only papers published on peer-reviewed journals were selected, and our search yielded 18 research articles.  Table 1 summarizes the literature from the used databases, the number (no.) of medical images in lesion classification and the diagnosis performance (AUC, the area under the curve; ACC, accuracy; SEN, sensitivity; SPE, specificity). Note that in each literature, only the model which achieves the best classification performance is reported.

### 3.2. Involved Databases

Table 1 indicates that mostly used mammography databases come from in-house collection (7/18), followed by public databases of BCDR-F03 (5/18), DDSM (4/18), INbreast (3/18), MIAS (1/18), and IRMA (1/18), and the last one comes from the DREAM challenge (1/18). The number of medical images in databases ranges mainly from several hundreds to thousands. Notably, the DREAM challenge is consisted of 82,000 images. Moreover, among the public databases, BCDR-F03 is the only one consisted of FM images, while among in-house collections, [55] is the one study that makes use of FM images (1655 FM images and 799 DM images), and all other databases and in-house collections are made up of DM images.

Three public databases of DDSM (http://marathon.csee.usf.edu/Mammography/Database.html), BCDR-F03 (http://bcdr.inegi.up.pt), INbreast (http://medicalresearch.inescporto.pt/breastresearch/index.php), and MIAS (http://peipa.essex.ac.uk/info/mias.html) are accessible online, while the DREAM challenge (https://www.synapse.org/#Synapse:syn4224222) is devoted to online competition and aims at improving the predictive accuracy of mammographic images for early detection and diagnosis of breast cancer. The IRMA [69] contains image patches selected from the DDSM, MIAS, and other two data sets. Among the public databases, DDSM (“Digital Database for Screening Mammography”) remains the largest available resource for mammographic image analysis [70]. It consists of 14 volumes of benign lesion cases and 15 volumes of malignant lesion cases in addition to 2 volumes of benign lesion cases without callback. It also contains 12 volumes of normal cases. The images in DDSM are in an outdated image format with a bit depth of 12 or 16 bits per pixel, and image resolution is larger than [4000, 3000], both depending on scanners.

The database BCDR-F03 (“Film Mammography Dataset Number 3”) is a subset of Breast Cancer Digital Repository (BCDR) that collects patient cases from the northern region of Portugal. It was made available for the development and comparison of algorithms [52]. The BCDR-F03 contains 344 patient cases, 736 FM images, and 406 breast lesions. Among the lesions, 230 are benign (426 images) and 176 malignant (310 images). Notably, BCDR-F03 contains FM images in the gray-level digitized TIFF (Tagged Image File Format) with a bit depth of 8 bits per pixel, and image resolution is [720, 1168].

The database INbreast is made up of 115 breast lesion cases and 410 digital images [71]. However, only 56 cases are histologically verified (11 benign and 45 malignant lesions). The mammographic images are saved in DICOM (Digital Imaging and Communications in Medicine) format with 14-bit contrast resolution. The image matrix is [2560, 2238] or [3328, 4084] depending on imaging scanners.

The MIAS database (“Mammographic Image Analysis Society”) contains 322 digital images among which 67 lesions are benign and 53 lesions are malignant [72]. Quite different from above-mentioned databases, MIAS provides the image coordinates of center of each abnormality and the approximate radius (in pixels) of a circle to enclose the abnormality, but not the coordinates of points localized on the boundary of lesions. Images are stored 8 bits per pixel in the PGM (Probabilistic graphical model) format. The database has been reduced to a 200 micron pixel edge and padded/clipped so that the image matrix is [1024, 1024].

### 3.3. Performance Metrics

To quantify the classification performance of CAD models, widely used metrics are AUC and ACC, followed by SEN and SPE (Table 1). Specifically, ACC, SEN, and SPE are computed based on the confusion matrix. As shown in Table 2, TP is the case which is histologically verified positive and correctly predicted as “positive”, while FN represents the case histologically verified positive but misclassified as “negative”. Furthermore, TN is the true negative case predicted correctly, and FP is the true negative case but predicted as “positive” [73]. Generally, benign lesions are labeled with “negative” and malignant lesions are labeled “positive”.

Given the labels and corresponding prediction results, ACC, SEN, and SPE can be, respectively, formulated as (TP + TN)/(TP + FN + FP + TN), TP/(TP + FN), and TN/(TN + FP). As to AUC, it is quantified based on the receiver operating characteristics (ROC) curve. ROC is a curve of probability and AUC presents a model's capacity of lesion differentiation. To these 4 performance metrics, higher values indicate better performance.

### 3.4. CNN-Based MBCD Models

In general, CNN-based models can be divided into dedicated models and transferred models. The former include the proposal of new architectures, the modification or integration of existing CNN models, while the latter make the most use of pretrained models and further fine-tune them by using medical instances. Furthermore, it is found that some models just use CNN for feature extraction and lesion diagnosis is fulfilled by using machine learning classifiers. In particular, handcrafted features are taken into consideration. Therefore, in this study, CNN-based MBCD models are broadly categorized into three groups of dedicated models, transferred models, and hybrid models.  Table 3 summarizes the CNN-based models from the model building to its pros and cons analysis. Note that the pros of “parameter initialization” indicate the model is pretrained with ImageNet.

#### 3.4.1. Dedicated MBCD Models

To enhance the diagnosis with unlabeled data, [54] proposes a graph-based semisupervised learning scheme, which is consisted of iterative data weighting, feature selection, and data labeling before using the modified LeNet for lesion diagnosis. Experimental results indicate that the scheme requires quite a small portion of labeled data (100 lesions) for training and achieves promising performance on the unlabeled data (3058 lesions). In addition, the scheme seems less sensitive to the initial labeled data. Reference [55] adds 2 fully connected layers at the last full-connection layer of the frozen AlexNet. The parameters in the AlexNet are initialized on the ImageNet and keep unchanged, while the whole model is trained on medical instances. Reference [58] proposes a four-layered model (3 convolutional layers and 1 full-connection layer) and a 4-fold cross-validation strategy is performed on 560 lesions (280 benign and 280 malignant). Reference [62] designs a CNN architecture (5 convolutional layers and 2 full-connection layers), while it pretrains the model on the ImageNet. Notably, parasitic metric learning is embedded that makes the best use of misclassified medical instances and improves the diagnosis performance. Reference [65] employs YOLO for lesion detection and localization followed by a tensor structure for the malignancy prediction. And consequently, automatic detection and classification of suspicious lesions is achieved simultaneously. Similarly, [66] uses the faster R-CNN for lesion detection and localization and the VGG for cancer diagnosis. The model is first trained on the DDSM and further validated on the INbreast and the DREAM challenge. It performs as one of the best approaches in mammographic image analysis. Reference [67] develops a hybrid model. It first uses the pretrained GoogLeNet for feature extraction, and 3072 features are obtained. And then, an attention mechanism is proposed for feature selection. At last, it uses LSTM to integrate both contexture information from multiview image features and information of clinical data for the lesion classification.

**Figure 4:**
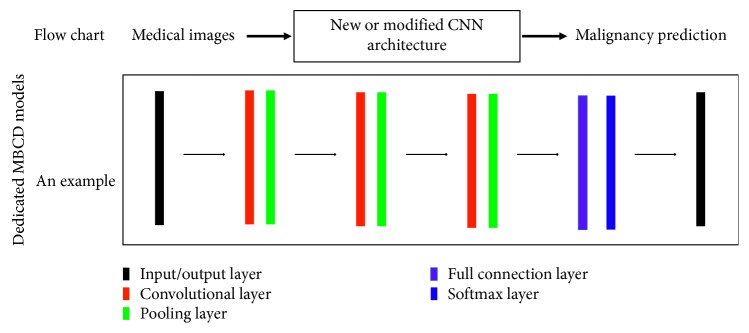
The flow chart and an example of dedicated MBCD models. The flow chart highlights the CNN is a newly designed or modified network, and the example describes the architecture of a CNN model in [58]. It should be noted that parameters of dedicated models are with random initialization followed by iterative optimization with medical instances.

Figure 4 demonstrates the flow chart and an example of dedicated MBCD models. The flow chart highlights that the CNN is a newly designed or modified network and the example describes the architecture of the CNN model from [58]. It should be noted that parameters of dedicated models are with random initialization followed by iterative optimization with medical instances.

Although [55, 62, 66, 67] make use of the ImageNet for parameter initialization, it should be highlighted that one develops a new architecture [62], one modifies the existing architecture and introduces a new learning strategy [55], and the others emphasize on the integration of two kinds of network architectures for simultaneous detection and localization and final lesion diagnosis [66, 67]. Therefore, [55, 62, 66, 67] are categorized into the group of dedicated models.

#### 3.4.2. Transferred CNN Models

Due to insufficient medical instances, deep CNN models pretrained on a large-scale of labeled natural images (such as ImageNet) are transferred and also fine-tuned with medical instances before the application in breast cancer diagnosis. Reference [61] gives out a systematic comparison of one shallow network (3 convolutional layers and 2 full-connection layers) and the AlexNet. Transfer learning is concerned, and experiment results indicate that CNN models with transfer learning outperform the models without transfer learning. Reference [63] investigates three kinds of implementation of an 8-layered CNN architecture. Parameters, such as the number of convolutional filters in each layer, are fine-tuned with mammographic lesion instances. Experimental comparison further indicates that incorporating handcrafted features increases the classification performance. Reference [64] concentrates the study on three deep learning models (VGG, RestNet, and GoogLeNet) and knowledge transfer is explored. Experiments are conducted to compare the random initialization and parameter initialization and to figure out how to fine-tune the models. Notably, three public databases (DDSM, INbreast and MIAS) are analyzed. Reference [68] compares two deep networks (AlexNet and GoogLeNet) which are pretrained on the ImageNet, two shallow CNN models, and two ML-based MBCD models. Experimental results suggest that knowledge transfer is helpful in breast lesion diagnosis.

**Figure 5:**
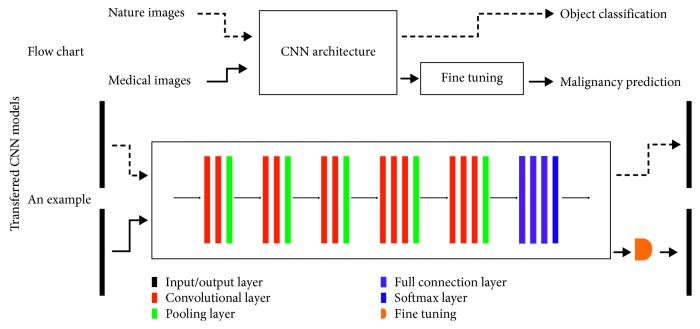
The flow chart and an example of transferred MBCD models. The flow chart emphasizes transfer learning (dashed arrows) and fine-tuning, and the example comes from [64] which makes use of pretrained VGG16 for malignancy prediction. It should be noted that parameters of pretrained models are well-determined in the source domain, while fine-tuning attempts to use medical instances for further optimization of these parameters toward the target task.

Figure 5 shows the flow chart and an example of transferred MBCD models. The flow chart highlights the offline training of a CNN model on nonmedical images, and moreover, it emphasizes fine-tuning the pretrained model with medical instances. A representative example using VGG as the diagnosis model comes from [64]. It should be noted that parameters of CNN architectures are predetermined in the task of object recognition, and their values are further optimized toward mammographic breast lesion differentiation.

Existing deep architectures are made the most use of, and these models [61, 63, 64, 68] are pretrained on the ImageNet and parameters are initialized. And then, mammographic lesion instances are used to fine-tune the deep models. While to further improve the diagnosis performance, additional techniques, such as data augmentation, are embedded in the training procedure. It should be noted that [61] has designed shallow networks, while its purpose is to verify whether transfer learning could improve the cancer diagnosis, and thereby it is grouped into the transferred CNN models.

#### 3.4.3. CNN Models as Feature Extractors

Among the CNN-based MBCD models, 7 out of 18 take CNN to retrieve high-level features for lesion representation. Reference [51] develops an 8-layered network (5 convolutional layers and 3 full-connection layers). The model is pretrained on the ImageNet to overcome the issue of limited medical instances. And then, SVM performs as the classifier and a decision mechanism is provided. After that, the MBCD model integrates 256 midlevel and 2048 high-level features for lesion classification. Reference [52] designs two shallow networks and experimental results indicate the 3-layered network (2 convolutional layers and 1 full-connection layer) obtains better performance. While for higher accuracy, SVM is further employed which takes these CNN features as its input. Experiment results show the diagnosis performance achieves slight but significant improvement when 17 low-level and 400 high-level features are pooled for lesion quantification. Reference [53] takes advantage of the pretrained AlexNet for the lesion differentiation. More specially, one SVM-based model uses 3795 high-level features as its input and the other SVM-based model uses 29 low-level features for the lesion classification. The outputs are fused by soft voting and significant improvements are achieved in malignancy prediction. Reference [56] investigates different methodologies for feature fusion. It concerns 38 handcrafted features and 1472 CNN learned features, and SVM is as the classifier for each kind of feature. Then, the results from each SVM are fused for final decision making. The results show that the integration of low- and high-level features significantly improves cancer diagnosis. Reference [57] proposes a hybrid framework for mammographic image analysis. With minimal user intervention, it is capable of mass detection, lesion segmentation, and malignancy prediction. Specifically, for lesion differentiation, it regresses the output of the CNN model to 781 handcrafted features and then, a full-connection layer is added for feature abstraction. Finally, RF is utilized to improve the diagnosis accuracy. Reference [59] introduces a shallow network (2 convolutional layers and 1 full-connection layer). It alternatively cooperates with discrete wavelet transform and curvelet transform for image preprocessing. At last, a total of 784 features are handcrafted. Moreover, both softmax and SVM are compared, and SVM outperforms softmax with slight increase. Reference [60] takes advantage of 1472 high-level features from the pretrained VGG with frozen parameters. Its novelty comes from the proposal of step-wise feature selection and the 2 most frequently selected features are used for SVM-based breast lesion classification.

**Figure 6:**
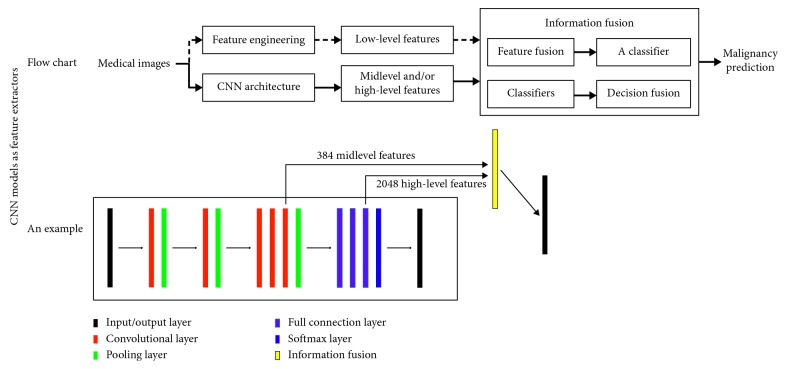
The flow chart and an example of CNN performing as feature extractors. The flow chart highlights the information fusion which can be further divided into two approaches, feature fusion followed by a classifier or decision fusion of lesion malignancy predicted by using one or more classifiers. The example comes from [51] which develops a new CNN model and the model is pretrained on ImageNet. At last, the model fuses the prediction results from SVM classifiers which separately use 384 midlevel features and 2014 high-level features as its input.

Figure 6 shows the flow chart and an example of CNN models as feature extractors. The flow chart highlights information fusion. In other words, whether a CNN model is newly designed or pretrained becomes not important and using low-level feature is optional. Information fusion can be divided into two approaches. One is feature fusion followed by a classifier, and the other is decision fusion of lesion malignancy predicted by using one or more classifiers. The example comes from [51] which develops a new CNN model and the model is pretrained on ImageNet. At last, the model fuses the prediction results (decision fusion) from SVM classifiers which separately use 384 midlevel features and 2048 high-level features as the input.

Prior studies have proved the benefits of low-level features in mammographic image analysis. And at present, how to select the informative CNN features [60] and how to fuse low-, mid-, and high-level features and clinical information have become important [52, 53, 56]. It should be mentioned that even if some MBCD models concern handcrafted features [53, 56], the ultimate purpose is to construct a hybrid framework for improved diagnosis and thereby, these publications [53, 56] are categorized into the third group.

#### 3.4.4. Technical Highlights among CNN-Based MBCD Models

Table 4 summarizes the technical highlights that can distinguish each kind of CNN-based MBCD models. In the Table, “✔” indicates the distinct component in the model, “✖” denotes the component is not included in the models, while “—” means the component is not important in this kind of CNN-based models.

## 4. Discussion

A total of 18 peer-reviewed journal publications (Table 1) are found with regard to the “convolutional neural network” or “deep learning” based “breast cancer diagnosis” using “mammography” images. The models are generally divided into three groups (Table 4): one highlights the design of new architectures or the modification or integration of existing networks (Figure 4); one concentrates on the use of transfer learning and fine-tuning in breast cancer diagnosis (Figure 5); and the last one concerns a hybrid model in which CNN performs for feature extraction and information fusion becomes indispensable in decision making (Figure 6). In addition, Table 3 summarizes these models from the model building to its pros and cons analysis.

### 4.1. Our Findings

To overcome the issue of limited medical instances, there are 10 publications that employ transfer learning [51, 53, 55, 56, 61–64, 66, 68], with or without fine-tuning. Transfer learning is able to alleviate this issue to some extent, since deep models have been optimized using massive amount of data in the source domain; and consequently, the time and labor can be considerably reduced in the target domain. In particular, it has been verified that transfer learning benefits the differentiation of breast lesions seen in mammographic images. Besides, to increase the number of medical instances, data augmentation is used [59, 61, 65, 68]. It makes sense in lesion malignancy prediction, since a lesion might be presented in any particular orientation in screening and thus, the MBCD model should be able to learn and recognize the lesion malignancy. For data augmentation, besides image rotation and flipping, other techniques can be adapted, such as image quality degrading (https://github.com/aleju/imgaug) and image deformation [74–76].

To improve the diagnosis performance, 11 out of 18 publications develop shallow architectures or modify existing networks [51, 52, 54, 57–60, 62, 65–67]. Shallow architectures decrease the number of medical instances for training, while machine learning classifiers should be utilized when modified deep networks with frozen or fine-tuned parameters perform as feature extractors. However, problems occur. The first problem concerns which classifier to be applied for the differentiation of benign and malignant lesions. It is found that 9 out of 11 publications select SVM [51, 52, 54, 58–60, 62, 65, 66], and 1 uses RF [57] and 1 chooses LSTM [67] for malignancy prediction. The second one is how to choose informative and predictive features among hundreds to thousands of variables. Most publications address this question by comprehensive experiments to make a trade-off between the diagnosis efficiency and effectiveness, while only [56] proposes using the frequency of the CNN feature selected in the training stage as the weighting of the feature importance. Last but not the least, it is time-consuming and troublesome. In general, it takes days to weeks to develop new architectures and to modify or to integrate deep models due to the requirements of model training, parameter optimization, feature selection, and algorithm comparison.

It is also found that 7 publications consider low-level and/or clinical features [51–54, 56, 59, 67]. Low-level features are mainly derived from intensity statistics, shape description, and texture analysis [34]. Specifically, these features can be further analyzed with multiscale decomposition or in transform spaces. Clinical information includes breast density, patients' age, and other symptoms, such as microcalcification. In addition, 4 publications provide the comparison between CNN- and ML-based models [51, 52, 56, 68] and ML-based models are treated as the baseline. It should be noted that ML-based models benefit from the prior knowledge and clinical experience in feature crafting, feature selection, and the use of machine learning classifiers. In particular, it is feasible to build a ML-based model on a very small database [36]. Besides, ML-based models are relatively lightweight computing and require no specific hardware and thus, these models can be easily deployed and managed in daily work.

Integrating multiple representation of mammographic lesions can enhance the performance of breast cancer diagnosis, while how to incorporate low-, mid-, and high-level features or multiview data is quite difficult. There are 4 publications [51, 53, 56, 67] which provide mechanism for information fusion or decision fusion. Reference [51] proposes a decision mechanism by evaluating the consistency of the results from the midlevel features and the high-level features. If not consistent, gray information would be added to assess the similarity and support the decision making. Both [53, 56] build ensemble classifiers by averaging the results from two SVM classifiers among which one makes use the pretrained CNN features and the other analyzes handcrafted features. Reference [67] utilizes LSTM cells to integrate the features from multiview data. Since multiview data contain contextual information, the variations among multiview images may contribute additional information in lesion interpretation.

### 4.2. Technical Challenges

Several technical challenges remain. The first challenge comes from how to use the pretrained deep CNN models which is closely related to the MBCD performance [77, 78]. However, there is no definitive answer on how to fine-tune the network and how many medical instances is sufficient for the fine-tuning, even good practice is available [79]. The simplest way is to take the parameters of the whole network or some layers of the network tunable. Some studies suggest layer-wise fine-tuning, while the time consumption will be dramatically increased [80]. On the other hand, when using deep models as feature extractors, other technical issues arise, including how to select high-level features, how to integrate multiperspective information, and which machine learning classifier is employed. It is pitiful that no tutorial or practical guidelines are repeatable. In clinic, to improve the performance of breast cancer diagnosis, various imaging modalities and clinical data are taken into account that further imposes difficulties on information fusion [9]. Since no one-size-fits-all solution is available, prior knowledge, previous studies, and empirical experience become more and more important to address these technical issues [78–83].

It is also challenging on how to avoid overfitting in the optimization of deep networks. Dropout is proposed to address the problem [84] which aims to randomly drop units (along with connections) from the network in the training stage. It can prevent units from coadapting too much, and a practical guide is provided for the training of a Dropout network [84]. It is full of potential to avoid overfitting by increasing the number of medical instances for training. At last, if there is no possibility to reduce the architecture complexity and no way to increase the number of training instances, the mainstream is to manipulate parameters, such as the learning rate, and to monitor the drop of performance metrics between the training phase and the validation phase [58, 60, 61, 68]. It also should be mentioned that the threshold of the drop is subjective, and thus, comprehensive experiments become necessary.

The third challenge is the curse of dimensionality [85]. It is known that the primary purpose of deep learning is for recognizing the target from thousands of object categories. However, MBCD is a binary classification problem, and the lesions seen in mammographic images are to be labeled as benign or malignant. Thus, it seems not convincing to use thousands of features for a binary classification problem regarding hundreds of medical instances [51–53, 56]. Some studies take recourse to feature selection [60] and feature dimension reduction [54]. As to deep networks, the frequency of features selected in the training phase as a weighting factor of feature importance is meaningful [60].

In practice, challenges exist in each step of the building of CNN-based MBCD models. First, a number of factors influence the quality of mammographic imaging, such as the imaging scanner and reconstruction methods, and both breast compression and motion artifacts during image acquisition further degrade the imaging quality. Therefore, quantitative image quality assessment may be necessary [86]. Moreover, due to different shapes and margin of suspicious lesions and also ambiguous boundaries between lesions and surrounding tissues, the quality of lesion delineation is unstable, and thereby, the techniques for automatic mammographic breast lesion detection and segmentation are still in need of improvement [87]. In addition, evolutionary pruning of knowledge transfer of deep models that are pretrained on sufficient medical images is promising for mammographic breast lesion diagnosis because of the similar feature space [88]. Last but not the least, it is always desirable to build a seamless system to localize the suspicious lesions and give out the malignancy prediction simultaneously [65, 66].

### 4.3. Future Focus

Except for the technical challenges aforementioned, another three topics should be focused on in the future work. The first one is to collect sufficient high-quality mammographic instances. Due to the limited funding, scarce medical expertise, and privacy issues, there is no big leap in data sharing, in particular, the mammographic lesion images. At present, the DDSM remains the largest publicly available database as well as the first choice in large-scale mammographic image analysis [89]. While based on the fact that over 150 million mammographic examinations are performed worldwide per year, there is significant room for improvement in data collection and sharing. In particular, lack of imaging data restricts the development and upgrading of intelligent systems for personalized diagnosis, including but not limited to the design of deeper architectures, hyperparameter optimization, and the evaluation of generalization capacity. Fortunately, rapid progress is seen in the era of big data and many public databases have been released online, such as TCIA (http://www.cancerimagingarchive.net/), and various challenges are open, such as the DREAM challenge. With such a standardization, it will become easier to compare different approaches on the same problem of the same database and thereby pushing forward the techniques of CNN-based MBCD.

Another topic is about the interpretation of the learned CNN features. In contrast to handcrafted features with mathematical formalization and clear explanation, the interpretation of retrieved CNN features is quite poor. One way to tackle this issue is from qualitative understanding [55, 58] based on visualization. Reference [90] provides a technique for layer-wise feature visualization. In object recognition, the technique indicates that shallow layers typically represent the presence of edges, middle layers mainly detects motifs by spotting particular arrangements of fine structures, while deep layers attempt to assemble these motifs into a larger cluster to be a part of or the whole object [19, 58]. It should be admitted that the layer-wise visualization technique facilitates the visual perception and further understanding of what the networks have learned. Reference [91] analyzed the predicted results in two-dimensional space using t-distributed stochastic neighbor embedding (t-SNE). The t-SNE represents each object by a point in a scatter plot where nearby points denote similar objects and distant points indicate dissimilar objects. Therefore, a clear insight is provided into the underlying structure of malignancy prediction [55]. Quantitative interpretation of deep learning is ongoing. Reference [92] gives a geometric view to understand the success of deep learning. They claim that the fundamental principle attributing to the success is the manifold structure in data, and deep learning can learn the manifold and the probability distribution on it. Reference [93] provides theory on how to interpret the concept learned and the decision made by a deep model. It further discusses a number of questions in interpretability, technical challenges, and possible applications. The third topic is the translation of the clinical research of CNN-based MBCD into the decision supporting in clinical practice. There is no doubt that deep learning tools can provide valuable and accurate information for cancer diagnosis, while it is impossible to take the role and responsibility of clinicians. The fundamental role of a clinician in routine work is to collaborate with other team members, including physicians, technologists, nurses, therapists, and even patients [94]. Thus, before accepting these decision-supporting systems for daily use, it should provide profound understanding and visual interpretation of deep learning tools, not only the surpassing human-level performance.

Furthermore, one big step to use CNN-based MBCD models for clinical applications comes from the review and approval from the Food and Drug Administration (FDA). To date, several FDA-approved CAD systems have been in the market, such as the QVCAD system (QView Medical Inc, Los Altos, CA) that uses deep learning for automated 3D breast ultrasound analysis. With the increasing use of deep learning algorithms, more and more CNN-based CAD systems will be approved by the FDA. Basically, compelling properties, such as expert-level performance, robustness, and generalizability, should be guaranteed on different imaging devices. While from the perspective of long-term evolution, a global real-life application accounting for widespread geographic, ethic, and genetic variations should be considered. Therefore, there is still a long way ahead of the translation of deep learning tools from scientific research to clinical practice.

### 4.4. Limitations

There are several limitations. First, this review focuses on CNN for automated MBCD. For computer-aided MBCD, it can also be well tackled by using other CAD techniques, such as case retrieval [95–97] and breast density estimation [98, 99]. Moreover, this study concerns only mammography. For comprehensive disease analysis, other imaging modalities, such as ultrasound and magnetic resonance, should be taken into consideration [31]. Besides, this review is limited to two-dimensional image analysis, and many other medical tasks use CNN models to tackle volumetric images [100–102]. In particular, this study concerns only peer-reviewed journal publications that considerably reduces the number of publications for analysis and consequently, it might omit some high-quality CNN-based MBCD models [103–105]. In addition, some technical details, such as how to prepare medical instances for training, are not delivered in this review, while it should be kept in mind that each step is related to mammographic image analysis.

## 5. Conclusion

This study presents a technical review of the recent progress of CNN-based MBCD. It categorizes the techniques into three groups based on how to use CNN models. Furthermore, the findings from the model building to the pros and cons of each model are summarized. In addition, technical challenges, future focus, and limitations are pointed out. At present, the design and use of CNN-based MBCD is at its early stage and result-oriented. To the ultimate goal of using deep learning tools to facilitate clinical practice, there is still a long way ahead. This review benefits scientific researcher, industrial engineers, and those who are devoted to intelligent cancer diagnosis.

## Figures and Tables

**Table 1:** A summary of CNN based MBCD methods.

	Year	Database	No. of images	AUC	ACC	SPE	SEN
[51]	2016	DDSM	600		0.967		
[52]	2016	BCDR-F03	736	0.82 ± 0.03			
[53]	2016	In-house	607	0.86			

[54]	2017	In-house	3158	0.88	0.82	0.72	0.81
[55]	2017	In-house	2454	0.82 ± 0.02			
[56]	2017	In-house	245	0.86 ± 0.01			
[57]	2017	INbreast	115	0.91 ± 0.12	0.95 ± 0.05		
[58]	2017	In-house	560	0.79 ± 0.02			
[59]	2017	IRMA	2796	0.839	0.837	0.854	0.797

[60]	2018	In-house	78	0.81 ± 0.05			
[61]	2018	In-house	3290	0.7274			
[62]	2018	DDSM	600		0.974		
		MIAS	120		0.967		
[63]	2018	BCDR-F03	736	0.813			
[64]	2018	DDSM	5316	0.98	0.9735		
		BCDR-F03	600	0.96	0.9667		
		INbreast	200	0.97	0.9550		
[65]	2018	DDSM	600		0.97		
[66]	2018	DREAM	82,000	0.85			
		INbreast	115	0.95			
[67]	2018	BCDR-F03	736	0.891	0.852		
[68]	2018	BCDR-F03	736	0.88	0.81		

**Table 2:** Confusion matrix.

	Predicted positive	Predicted negative
Histologically verified positive	True positive (TP)	False negative (FN)
Histologically verified negative	False positive (FP)	True negative (TN)

**Table 3:** Summary of CNN-based MBCD models from the model building to its pros and cons analysis.

Publication (year)	Approach	Pros (+)/cons (−)
[51] (2016)	(1) An 8-layered CNN	+parameter initialization
	(2) SVM-based decision mechanism	+decision mechanism
	(3) Compared to ML- and CNN-based models	−256 mid- and 2048 high-level features

[52] (2016)	(1) A 3-layered CNN	+medical instances for training
	(2) SVM-based classification	−17 low- and 400 high-level features
	(3) Compared to ML- and CNN-based models	−a shallow CNN model

[53] (2016)	(1) Transferred AlexNet	+parameter initialization
	(2) SVM-based classification	+soft-voting-based decision mechanism
	(3) Classifier-based soft voting	−29 low- and 3795 high-level features

[54] (2017)	(1) Modified LeNet	+semisupervised learning
	(2) Graph based semisupervised learning	+a few labeled data used for training
	(3) Feature dimension reduction	+less sensitive to initial labeled data
	(4) Using unlabeled data	

[55] (2017)	(1) Modified AlexNet	+parameter initialization
	(2) Multitask transfer learning	+improved generalizability

[56] (2017)	(1) Transferred the VGG	+parameter initialization
	(2) SVM-based classification	+decision mechanism
	(3) Compared to ML- and CNN-based models	−38 low- and 1472 high-level features

[57] (2017)	(1) R-CNN for detection and diagnosis	+minimal user intervention in image analysis
	(2) Feature regression	−781 low-level features for CNN feature regression
	(3) RF-based classification	

[58] (2017)	(1) A 4-layered CNN	+medical instances for training
		−a shallow CNN model

[59] (2017)	(1) A 3-layered CNN	+medical instances for training
	(2) SVM-based classification	+image analysis in transformed domain
	(3) Data augmentation	−a shallow CNN model

[60] (2018)	(1) VGG for feature extraction	+2 features selected for diagnosis
	(2) Stepwise feature selection	
	(3) SVM-based classification	

[61] (2018)	(1) Transferred AlexNet	+parameter initialization
	(2) Data augmentation	
	(3) Compared to CNN models	

[62] (2018)	(1) A 7-layered CNN	+parameter initialization
	(2) Parasitic metric learning	+parasitic metric learning

[63] (2018)	(1) Transferred VGG	+parameter initialization
	(2) Compared to CNN-based models	

[64] (2018)	(1) Transferred VGG/ResNet/Inception	+parameter initialization
	(2) Comparison on 3 databases	+systematic comparison
		−time consuming

[65] (2018)	(1) YOLO and tensor structure	+medical instances for training
	(2) Data augmentation	+simultaneous detection and classification

[66] (2018)	(1) Faster R-CNN and VGG	+medical instances for training
	(2) Pretrained with the DDSM	+both detection and diagnosis
		+evaluated on a large-scale screening dataset

[67] (2018)	(1) GoogLeNet for feature extraction	+medical instances for training
	(2) Attention mechanism for feature selection	+multiview and clinical information fusion
	(3) LSTM for feature fusion	

[68] (2018)	(1) Transferred AlexNet/GoogLeNet	+parameter initialization
	(2) Data augmentation	
	(3) Compared to ML- and CNN-based models	

**Table 4:** Technical highlights.

	New architecture	Transfer learning	Fine-tuning	Information fusion
Dedicated CNN models	✔	✖	✖	✖
Transferred CNN models	—	✔	✔	✖
CNN models as feature extractors	—	—	—	✔
